# Supplementary material for: Research on the influence of the morphology of coarse aggregates from Reclaimed Asphalt Pavement (RAP) on the mechanical properties of asphalt concrete based on finite element modeling
Source: PLoS One. 2025 Sep 12;20(9):e0323128. doi: 10.1371/journal.pone.0323128 (PMC12431242; doi:10.1371/journal.pone.0323128)
Supplement: S1 Appendix — (DOCX) [file pone.0323128.s001.docx]

**Appendix**

from abaqus import *

from abaqusConstants import *

import random

import numpy as np

import time

import math

from shapely.geometry import Polygon # Polygon

import scipy.io as io

from shapely.geometry import box, Polygon

from shapely.ops import cascaded_union

#********************* original parameters*************************

yuanxin = (0,0)

Rz = 50.8

# aspect ratio control

aspectmin=1

aspectmax=2

# Edge Angle control

ANmin = 0.98

ANmax = 1

# Shape index control

FFImin = 0

FFImax = 1

# Roundness ratio control

CCRmin = 0

CCRmax = 1

SZ = pi * Rz**2

R = [19, 16, 13.2, 9.5, 4.75, 2.36]#[9.5, 8, 6.6, 4.75, 2.375, 1.18]

p = [1, 0.98, 0.95, 0.9,0.7,0]

sa=[]

sai = 0

saa = []#Required feeding area for each grade

for i in range(0,5):

sa.append( 3.14 * ((R[i] + R[i+1])/4)**2 )

for i in range(0,5):

sai+=(p[i]-p[i+1]) * 3.14 * ((R[i] + R[i+1])/4)**2

for i in range(0,5):

saa.append(SZ*0.6/sai * (p[i]-p[i+1]) * 3.14 * ((R[i] + R[i+1])/4)**2)

po = 0

po = SZ*0.003

#******************************************************

# part1

s1 = mdb.models['Model-1'].ConstrainedSketch(name='__profile__',

sheetSize=500.0)

g, v, d, c = s1.geometry, s1.vertices, s1.dimensions, s1.constraints

s1.setPrimaryObject(option=STANDALONE)

s1.CircleByCenterPerimeter(center=yuanxin, point1=(Rz, 0))

p = mdb.models['Model-1'].Part(name='Part-1', dimensionality=TWO_D_PLANAR,

type=DEFORMABLE_BODY)

p = mdb.models['Model-1'].parts['Part-1']

p.BaseShell(sketch=s1)

s1.unsetPrimaryObject()

p = mdb.models['Model-1'].parts['Part-1']

del mdb.models['Model-1'].sketches['__profile__']

s = mdb.models['Model-1'].ConstrainedSketch(name='__profile__',

sheetSize=500.0)

g, v, d, c = s.geometry, s.vertices, s.dimensions, s.constraints

s.setPrimaryObject(option=STANDALONE)

# Roundness ratio control function

def calculate_CCR(vertices):

polygon = Polygon(vertices)

area = polygon.area

convex_hull = polygon.convex_hull

convex_hull_area = convex_hull.area

CCR = area / convex_hull_area

if CCRmin < CCR < CCRmax:

return True

else:

return False

# Shape parameter control function

def shape_parameter(vertices):

P = 0

A = 0

n = len(vertices)

for i in range(n):

x1, y1 = vertices[i]

x2, y2 = vertices[(i+1)%n]

dx = x2 - x1

dy = y2 - y1

P += math.sqrt(dx*dx + dy*dy)

A += x1*y2 - x2*y1

A /= 2

if A < 0:

A = -A

FFI = 4 * math.pi * A / (P * P)

if FFImin < FFI < FFImax:

return True

else:

return False

# Multilateral diameter control function

def polygon_diameter(vertices, D):

vertices = sorted(vertices, key=lambda v: v[0])

diameter = 0

k = False

for i in range(len(vertices)):

for j in range(i+1, len(vertices)):

if vertices[j][0] - vertices[i][0] > diameter:

break

dist = math.sqrt((vertices[j][0]-vertices[i][0])**2 + (vertices[j][1]-vertices[i][1])**2)

if dist > diameter:

diameter = dist

if diameter > D:

k = True

return k

# Edge Angle control function

def calculate_AN(polygonn):

polygon = Polygon(polygonn).convex_hull

convex_hull = polygon.convex_hull

if not isinstance(convex_hull, Polygon):

return None

Pc = convex_hull.length

centroid = Point(polygon.centroid.x, polygon.centroid.y)

farthest_point_distance = 0

for point in polygon.exterior.coords:

point_distance = centroid.distance(Point(point[0], point[1]))

if point_distance > farthest_point_distance:

farthest_point_distance = point_distance

a = farthest_point_distance

b = math.sqrt(polygon.area / math.pi)

Pe = math.pi * (3 * (a + b) - math.sqrt((3 * a + b) * (a + 3 * b)))

AN = (Pc / Pe) ** 2

if ANmax > AN > ANmin:

return True

else:

return False

# Aspect ratio control function

def max_aspect_ratio(polygonn):

polygon = Polygon(polygonn).convex_hull

rect = polygon.minimum_rotated_rectangle

pts = list(rect.exterior.coords)

width = ((pts[0][0] - pts[1][0]) ** 2 + (pts[0][1] - pts[1][1]) ** 2) ** 0.5

height = ((pts[1][0] - pts[2][0]) ** 2 + (pts[1][1] - pts[2][1]) ** 2) ** 0.5

aspect_ratio = max(width / height, height / width)

if aspectmax > aspect_ratio > aspectmin:

return True

else:

return False

# Polygon area calculation function

def polygon_area(coords):

coords = coords + [coords[0]]

area = 0.0

for i in range(len(coords) - 1):

area += coords[i][0] * coords[i + 1][1] - coords[i + 1][0] * coords[i][1]

area = abs(area) / 2.0

return area

# Polygon interference function

def Cal_area_2poly(newone, polygon):

sign = True

poly1 = Polygon(newone).convex_hull

for each_polygon in polygon:

poly2 = Polygon(each_polygon).convex_hull

if poly1.distance(poly2) <= 0.3:

sign = False

break

return sign

center = []

polygon = []

sums = [0,0,0,0,0]

new_coordf=[]

count = 0

center1=[]

#Stage 1-3 aggregate delivery

for i in range(0,3):

while True:

x = random.uniform(-Rz, Rz)

y = random.uniform(-Rz, Rz)

if x**2 + y**2 <= (Rz-R[i]/2)**2:

edge_num = random.randint(8, 15)

edge_0 = random.uniform(0, 2 * pi)

angle = []

for j in range(edge_num):

pj = 2 * pi / (edge_num * 3)

angle.append(random.uniform(edge_0 + pj + 3 * j * pj, edge_0 + 3 * pj + 3 * j * pj))

coord = []

coordf = []

for each_angle in angle:

px = x + R[i + 1]/2 * cos(each_angle)

py = y + R[i + 1]/2 * sin(each_angle)

coord.append((px, py, each_angle))

coordf.append((px, py))

lmin = -(R[i]-R[i + 1])/2

lmax = (R[i]-R[i + 1])/2

for a in range(edge_num):

l = random.uniform(lmin, lmax)

px = coord[0][0] + l * cos(coord[0][2])

py = coord[0][1] + l * sin(coord[0][2])

new_coordf = [(px, py) if k == (coord[0][0], coord[0][1]) else k for k in coordf]

coordf = new_coordf

coord.remove(coord[0])

sign = Cal_area_2poly(new_coordf, polygon)

if calculate_CCR(new_coordf)* shape_parameter(new_coordf)*calculate_AN(new_coordf)*max_aspect_ratio(new_coordf)*polygon_diameter(new_coordf, R[i+1]):

if sign:

area=polygon_area(new_coordf)

sums[i] += area

polygon.append(new_coordf)

center1.append(new_coordf)

count = 0

for o in range(edge_num - 1):

s.Line(point1=new_coordf[o], point2=new_coordf[o + 1])

s.Line(point1=new_coordf[0], point2=new_coordf[-1])

print(sums)

print(saa)

else:

count += 1

else:

count += 0.001

if saa[i] - sa[i] / 2 <= sums[i] <= saa[i] + sa[i] / 2 or count >= 5000:

break

n = 0

# 4th aggregate release

for i in range(3,4):

while True:

x = random.uniform(-Rz, Rz)

y = random.uniform(-Rz, Rz)

if x**2 + y**2 <= (Rz-R[i]/2)**2:

edge_num = random.randint(8, 15)

edge_0 = random.uniform(0, 2 * pi)

angle = []

for j in range(edge_num):

pj = 2 * pi / (edge_num * 3)

angle.append(random.uniform(edge_0 + pj + 3 * j * pj, edge_0 + 3 * pj + 3 * j * pj))

coord = []

coordf = []

for each_angle in angle:

px = x + R[i + 1]/2 * cos(each_angle)

py = y + R[i + 1]/2 * sin(each_angle)

coord.append((px, py, each_angle))

coordf.append((px, py))

lmin = 0

lmax = (R[i]-R[i + 1])/2

for a in range(edge_num):

l = random.uniform(lmin, lmax)

px = coord[0][0] + l * cos(coord[0][2])

py = coord[0][1] + l * sin(coord[0][2])

new_coordf = [(px, py) if k == (coord[0][0], coord[0][1]) else k for k in coordf]

coordf = new_coordf

coord.remove(coord[0])

sign = Cal_area_2poly(new_coordf, polygon)

if calculate_CCR(new_coordf)* shape_parameter(new_coordf)*calculate_AN(new_coordf)*max_aspect_ratio(new_coordf)*polygon_diameter(new_coordf, R[i+1]):

if sign:

area=polygon_area(new_coordf)

sums[i] += area

polygon.append(new_coordf)

center1.append(new_coordf)

count = 0

for o in range(edge_num - 1):

s.Line(point1=new_coordf[o], point2=new_coordf[o + 1])

s.Line(point1=new_coordf[0], point2=new_coordf[-1])

print(sums)

print(saa)

else:

count += 1

else:

count += 0.001

if saa[i] - sa[i] / 2 <= sums[i] <= saa[i] + sa[i] / 2 or count >= 3000:

break

n = 0

# 5th aggregate release

for i in range(4,5):

while True:

x = random.uniform(-Rz, Rz)

y = random.uniform(-Rz, Rz)

if x**2 + y**2 <= (Rz-R[i]/2)**2:

edge_num = random.randint(8, 15)

edge_0 = random.uniform(0, 2 * pi)

angle = []

for j in range(edge_num):

pj = 2 * pi / (edge_num * 3)

angle.append(random.uniform(edge_0 + pj + 3 * j * pj, edge_0 + 3 * pj + 3 * j * pj))

coord = []

coordf = []

for each_angle in angle:

px = x + R[i + 1]/2 * cos(each_angle)

py = y + R[i + 1]/2 * sin(each_angle)

coord.append((px, py, each_angle))

coordf.append((px, py))

lmin = 0

lmax = (R[i]-R[i + 1])/2

for a in range(edge_num):

l = random.uniform(lmin,lmax)

px = coord[0][0] + l * cos(coord[0][2])

py = coord[0][1] + l * sin(coord[0][2])

new_coordf = [(px, py) if k == (coord[0][0], coord[0][1]) else k for k in coordf]

coordf = new_coordf

coord.remove(coord[0])

sign = Cal_area_2poly(new_coordf, polygon)

if calculate_CCR(new_coordf)* shape_parameter(new_coordf)*calculate_AN(new_coordf)*max_aspect_ratio(new_coordf)*polygon_diameter(new_coordf, R[i+1]):

if sign:

area=polygon_area(new_coordf)

sums[i] += area

polygon.append(new_coordf)

count = 0

for o in range(edge_num - 1):

s.Line(point1=new_coordf[o], point2=new_coordf[o + 1])

s.Line(point1=new_coordf[0], point2=new_coordf[-1])

print(sums)

print(saa)

else:

count += 1

else:

count += 0.001

if saa[i] - sa[i] / 2 <= sums[i] <= saa[i] + sa[i] / 2 or count >= 3000:

break

#part2

p = mdb.models['Model-1'].Part(name='Part-2', dimensionality=TWO_D_PLANAR,

type=DEFORMABLE_BODY)

p = mdb.models['Model-1'].parts['Part-2']

p.BaseShell(sketch=s)

s.unsetPrimaryObject()

p = mdb.models['Model-1'].parts['Part-2']

del mdb.models['Model-1'].sketches['__profile__']

#part3

a = mdb.models['Model-1'].rootAssembly

a = mdb.models['Model-1'].rootAssembly

p = mdb.models['Model-1'].parts['Part-1']

a.Instance(name='Part-1-1', part=p, dependent=ON)

p = mdb.models['Model-1'].parts['Part-2']

a.Instance(name='Part-2-1', part=p, dependent=ON)

a = mdb.models['Model-1'].rootAssembly

a.InstanceFromBooleanMerge(name='Part-3', instances=(a.instances['Part-1-1'],

a.instances['Part-2-1'], ), keepIntersections=ON,

originalInstances=SUPPRESS, domain=GEOMETRY)

s = mdb.models['Model-1'].ConstrainedSketch(name='__profile__',

sheetSize=500.0)

g, v, d, c = s.geometry, s.vertices, s.dimensions, s.constraints

s.setPrimaryObject(option=STANDALONE)

spo=0
