# Supplementary material for: Research on the influence of the morphology of coarse aggregates from Reclaimed Asphalt Pavement (RAP) on the mechanical properties of asphalt concrete based on finite element modeling
Source: PLoS One. 2025 Sep 12;20(9):e0323128. doi: 10.1371/journal.pone.0323128 (PMC12431242; doi:10.1371/journal.pone.0323128)
Supplement: Attachment — Submitted filename: comments.docx [file pone.0323128.s002.docx]

Comments to the Author

I appreciate the opportunity to review this interesting manuscript entitled“Research on the Influence of the Coarse Aggregates’ Morphology on the Splitting Performance of Asphalt Mixtures Based on Finite Element Modeling”. In this paper, through finite element modeling, the morphology of coarse aggregate is effectively reflected, and the influence of aggregate morphology on the cracking resistance of asphalt mixture is accurately characterized. Generally, this paper needs some minor revisions before it is accepted for publication. The detailed comments are as follows.

1. Abstract is well-structured, but several areas can be improved for clarity, statistical significance to strengthen the findings, minor revisions for clarity, and emphasis on the novelty and potential applications.
2. The research background part lacks clear logical stratification. It can be classified and expounded according to research methods or research emphases to enhance the organization of paragraphs.
3. It is suggested that more details of the comparison between indoor test and model results should be supplemented to enhance the credibility of the model.
4. It is recommended that some references should be cited in the results and discussion part to improve the credibility of the analysis.
5. The potential significance and future perspective of this study can be mentioned in the conclusion.
6. English needs editing and revision. Some sentences are not concise and fluent, such as some long sentences with complex structures. It is suggested that the language of the paper should be polished to improve the conciseness and fluency of the expression.
7. Cite more references of recent years.
