# Supplementary material for: Research on the influence of the morphology of coarse aggregates from Reclaimed Asphalt Pavement (RAP) on the mechanical properties of asphalt concrete based on finite element modeling
Source: PLoS One. 2025 Sep 12;20(9):e0323128. doi: 10.1371/journal.pone.0323128 (PMC12431242; doi:10.1371/journal.pone.0323128)
Supplement: Attachment — Submitted filename: renamed_f7955.docx [file pone.0323128.s004.docx]

Response to ReviewerⅢComments

**Comments 1:**  Complete review of English – adjust verb tenses.

**Response 1**: Thank you for pointing this out. I have combed through and corrected the grammar in the article.

**Comments 2:** Correct the font numbering in the text, placing it in ascending order.

Response 2: Agree. I have revised the numbers of the references.

**Comments 3:**  The introduction must include the most current references, preferably from the last 5 years.

**Response 3**: Thank you for pointing this out, I have revised the introduction anew and updated the references.

**Comments 4:**  The article validates the accuracy of a two-dimensional finite element microscopic asphalt concrete model through an indoor Marshall splitting test. However, relying solely on this single test method may not provide a comprehensive overview, and it is advised to incorporate additional mechanical property tests.

**Response 4**: Thank you for your suggestions. I have added the compressive strength test back into the article and analyzed the shape parameters of the recycled coarse aggregates in combination with the splitting tensile test.

**Comments 5:** The research primarily utilizes a two-dimensional model, which showcases the mechanical properties of asphalt mixtures to some extent. Nevertheless, there remain discrepancies when compared to the real three-dimensional structure. Establishing a three-dimensional digital specimen model is recommended to more accurately simulate the internal structure and mechanical behavior of asphalt mixtures.

**Response 5**: Thank you for pointing this out. Since the method employed in this paper is somewhat innovative, and two more shape characteristics of aggregates have been newly added in this revision to characterize.

**Comments 6:**  While the effects of selected aspect ratio and shape parameters on splitting performance have been analyzed, there has been insufficient exploration of the sensitivity of these parameters. Furthermore, other morphological characteristics of aggregates could also influence the performance of asphalt mixtures. It is proposed to introduce additional parameters related to the morphological characteristics of aggregates to perform a more comprehensive analysis of their synergistic effects on asphalt mixture performance.

**Response 6**: Thank you for your suggestions. Besides the aspect ratio and shape parameter, I have added the roundness ratio and angularity to further analyze the influence of aggregate shape parameters on the mechanical properties of asphalt concrete.
